# Supplementary material for: Topical Delivery of Diacetyl Boldine in a Microemulsion Formulation for Chemoprotection against Melanoma
Source: Pharmaceutics. 2023 Mar 10;15(3):901. doi: 10.3390/pharmaceutics15030901 (PMC10054442; doi:10.3390/pharmaceutics15030901)

Supplementary Materials

Table S1. Mathematical models for *in vitro* release of microemulsion formulations of MCT-DAB, F1 and F2. R^2 values for the fitted release results for various mathematical models.

**Formulation**	**Zero-order (r^2)**	**First-order (r^2)**	**Higuchi (r^2)**	**Hixson Crowell (r^2)**	**Korsmeyer Peppas (r^2)**
MCT-DAB	0.9387	0.8105	0.9926	0.8605	0.9885
F1	0.9617	0.8230	0.9967	0.8795	0.9907
F2	0.9904	0.8789	0.9971	0.9327	0.9961

Figure S1:

Particle size distribution curves for various formulations. a = ME1; b = ME2; c = F1; d = F2

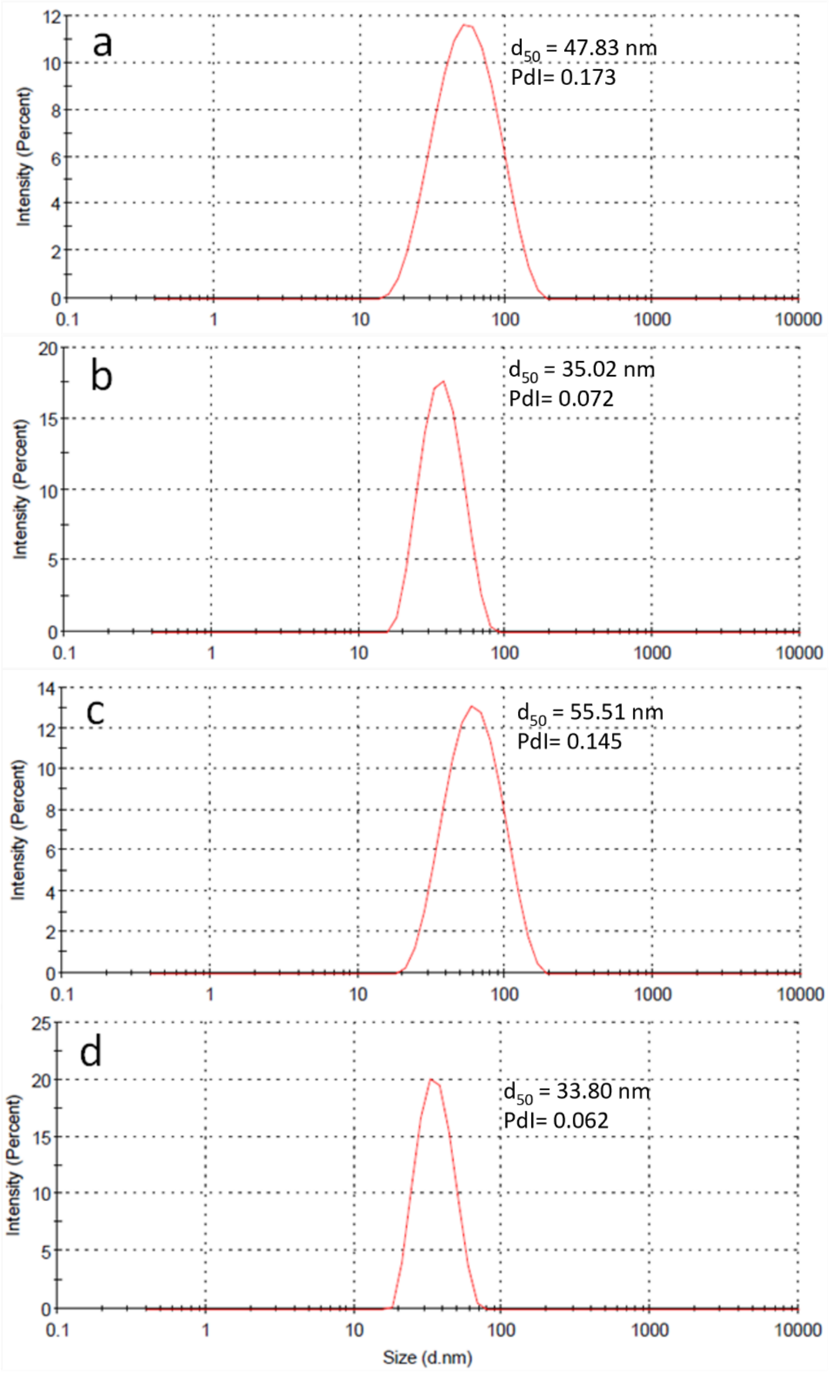

Supplement: Supplementary file 1 [file pharmaceutics-15-00901-s001.zip › pharmaceutics-2035574-supplementary.pdf]
